# Going beyond the Ordered Bulk: A Perspective on the Use of the Cambridge Structural Database for Predictive Materials Design

**DOI:** 10.1021/acs.cgd.4c00694

**Published:** 2024-08-19

**Authors:** Ioanna Pallikara, Jonathan M. Skelton, Lauren E. Hatcher, Anuradha R. Pallipurath

**Affiliations:** † School of Chemical and Process Engineering, University of Leeds, Leeds LS2 9JT, U.K.; ‡ Department of Chemistry, University of Manchester, Manchester M13 9PL, U.K.; § School of Chemistry, Cardiff University, Cardiff CF10 3AT, U.K.

## Abstract

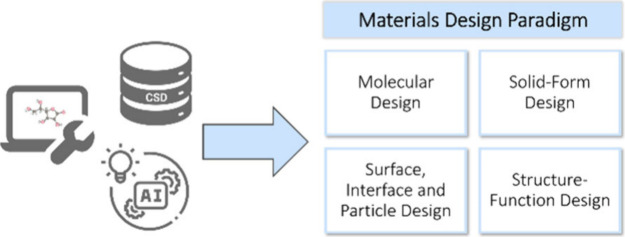

When Olga Kennard founded the Cambridge Crystallographic Data Centre in 1965, the Cambridge Structural Database was a pioneering attempt to collect scientific data in a standard format. Since then, it has evolved into an indispensable resource in contemporary molecular materials science, with over 1.25 million structures and comprehensive software tools for searching, visualizing and analyzing the data. In this perspective, we discuss the use of the CSD and CCDC tools to address the multiscale challenge of predictive materials design. We provide an overview of the core capabilities of the CSD and CCDC software and demonstrate their application to a range of materials design problems with recent case studies drawn from topical research areas, focusing in particular on the use of data mining and machine learning techniques. We also identify several challenges that can be addressed with existing capabilities or through new capabilities with varying levels of development effort.

## Abstract 2

We provide a perspective on the use of the Cambridge Structural Database for predictive design of molecular materials, highlighting case studies drawn from topical research areas and identifying opportunities for the future.

## 1 Introduction

In the age of “big data”, one can only marvel at the foresight Dr Olga Kennard had when she established the Cambridge Crystallographic Data Centre (CCDC) in 1965.^1^ The Cambridge Structural Database (CSD) marks one of the pioneering attempts to capture scientific data in a standard format, with the vision of leveraging vast quantities of data to learn new things. More than half a century later, with over 1.25 million structures, the CSD has not only stood the test of time but remains at the forefront of contemporary data-driven materials science.

Depositing small-molecule crystal structures with the CSD is standard practice and a requirement for most academic journals, ensuring that the database is continually updated both with new structures and more accurate determinations of known structures. The CCDC also develops and maintains a collection of software tools to enable the community to leverage the CSD for their research. Such tools include the graphical interface (GUI) for searching and retrieval, ConQuest,^2^ the visualization and analysis program, Mercury,^3^ and a Python application programming interface (API). In 2009, a consortium of industries, the Crystal Form Consortium, was founded to drive forward solid-form analysis and development, which led to the creation of the CSD-Materials software suite of tools for solid form analysis.^2−4^ The CSD-Discovery suite was similarly developed to include tools for computer-aided drug discovery.^5,6^

### 1.1 Molecular Materials Design Approaches

The foundational data and software tools provided by the CSD provides a powerful platform for the design of crystalline molecular materials, a multiscale problem covering length scales from angstroms (Å) to millimeters (mm).

At molecular scale, researchers can exploit synthetic chemistry to target molecular properties such as color, magnetism and biological activity. At this stage, the consideration is primarily the functionalization of the molecule itself, rather than the form it is used in for its intended application. However, most materials are ultimately stabilized as a solid, and often in a thermodynamically stable, ordered crystalline state (e.g., for maximizing shelf life), which we refer to henceforth as the “solid-state”.^7^

At the level of the solid-state, controlling the 3D packing of molecules to form a crystal structure determines a number of physical properties, for example mechanical behavior and solubility.^7^ At this stage the issue of polymorphism arises, whereby the same molecule can form multiple crystal structures. The outcome of a crystallization can be controlled by varying environmental conditions such as the temperature, pressure, and polarity or pH of the medium. These can, for example, favor molecules adopting a particular conformation or charge state (e.g., the zwitterion forms of amino acids), leading to polymorphs with very different properties. A well-known example of polymorphism is 5-methyl-2-((2-nitrophenyl)amino)thiophene-3-carbonitrile, also known as “ROY” for its vivid red, orange and yellow polymorphs, which has 12 confirmed polymorphs and an additional one proposed but yet to be confirmed.^8,9^ Polymorphism in drugs and agrochemicals has important biological and economic consequences and is thus heavily researched. Another familiar example of solid-state design is the optimization of pore size and accommodation of guest molecules in metal–organic frameworks (MOFs), which have applications ranging from hydrogen storage^10^ to catalysis.^11^

Structure–function relationships can be exploited during solid-state design to create “functional” crystalline materials. Bringing two molecules together to form a cocrystal can dramatically change the physical properties, for example cocrystallizing two colorless molecules to produce a thermochromic cocrystal that changes color in response to temperature changes through charge transfer between the components.^12^

More challenging, but no less important, is control over the surfaces and interfaces during crystal growth. In the pharmaceutical industry, crystallization remains the technique of choice for separation and purification. The morphology of the crystalline particles is an important process engineering parameter, and the functional groups exposed at the surfaces determine how the particles interact with the environment. The industry is moving toward new modalities of therapeutics based on molecules with increasing size and flexibility, which inevitably results in more complex surface chemistry. Formulations based on nanoparticles are also an emergent interest, and the high surface to bulk ratios of these materials makes understanding the surface chemistry crucial to bringing them to market. Surface and interface engineering is also key to producing complex architectures, such as flexible electronics, where organic materials must interface to metals and semiconductors and need to be processed using methods compatible with existing semiconductor manufacturing processes.

Finally, materials design can also consider scale up and manufacturing. While these are often regarded as engineering problems and considered out of scope during the initial materials design phases, where obtaining the required functionality may take precedence, targeting certain physical properties early (e.g., solubility in a particular solvent, or a desirable particle morphology) can make subsequent scale up easier. Tools such as the COnductor like Screening MOdel for Real Solvents (COSMO-RS)^13^ can be used to probe the thermodynamics of chemical processes, with one example being the use of COSMO with the CCDC Molecular Complementarity tool^14^ to screen for potential cocrystals of the agrochemical pymetrozine to obtain solid forms with improved solubility and stability.^15^

### 1.2 Challenges to Molecular Materials Design

The design of molecular materials presents some unique challenges. Firstly, molecules can present multiple isomers, with functional groups in different relative positions, leading to different molecular properties. Secondly, isomerism, together with the inherent conformational freedom of organic molecules, can have a significant influence on crystal packing. These influences can occur both through the intermolecular interactions in the crystal, but also through the interactions with solvent molecules during crystallization from solution. Finally, the interaction of the molecules and crystal particles with the environment can result in different functional groups being exposed at the surface, which adds a further layer of complexity to designing surfaces and interfaces.

This inherent complexity has catalyzed the development of innovative, data-driven approaches, using data mining and machine learning (ML) techniques to relate chemical and structural descriptors to properties of interest. These methods depend critically on the availability of high-quality data sets and on tools to efficiently search the data and extract descriptors, which makes the CSD and the CCDC tools an incredibly valuable resource.

In this perspective, we explore the ways in which the CCDC has driven materials design forward, highlighting important contemporary challenges that can be addressed using the CSD and the CCDC software stack and identifying some opportunities for the future and potential approaches for exploiting them. The material is organized as follows. In Section 2, we briefly outline the two main approaches to data-driven materials design. In Sections 3–8, we then outline how the CSD and CCDC software suite can be used for each part of the materials design process outlined above, providing examples of recent case studies and highlighting areas for future development. Each of these topics are large fields in their own right, and we therefore necessarily prioritize breadth over depth and direct interested readers to other, more detailed reviews where appropriate. Finally, we finish with some concluding remarks in Section 8.

## 2 Data-Driven Approaches to Materials Design

Data-driven approaches to materials design offer several potential advantages over more established experimental and computational techniques. Data mining or ML techniques can be used to efficiently analyze vast chemical spaces, identify relevant structure–property relationships, and potentially even generate predictive models that generalize to predicting the properties of unseen materials.^16−18^ Applications of these techniques range from prioritizing candidate materials and reducing expensive or time-consuming experimental trials, to finding strategies to optimize known material for specific applications and identifying new materials with novel properties or functionality.^18−21^

**Figure 1:**
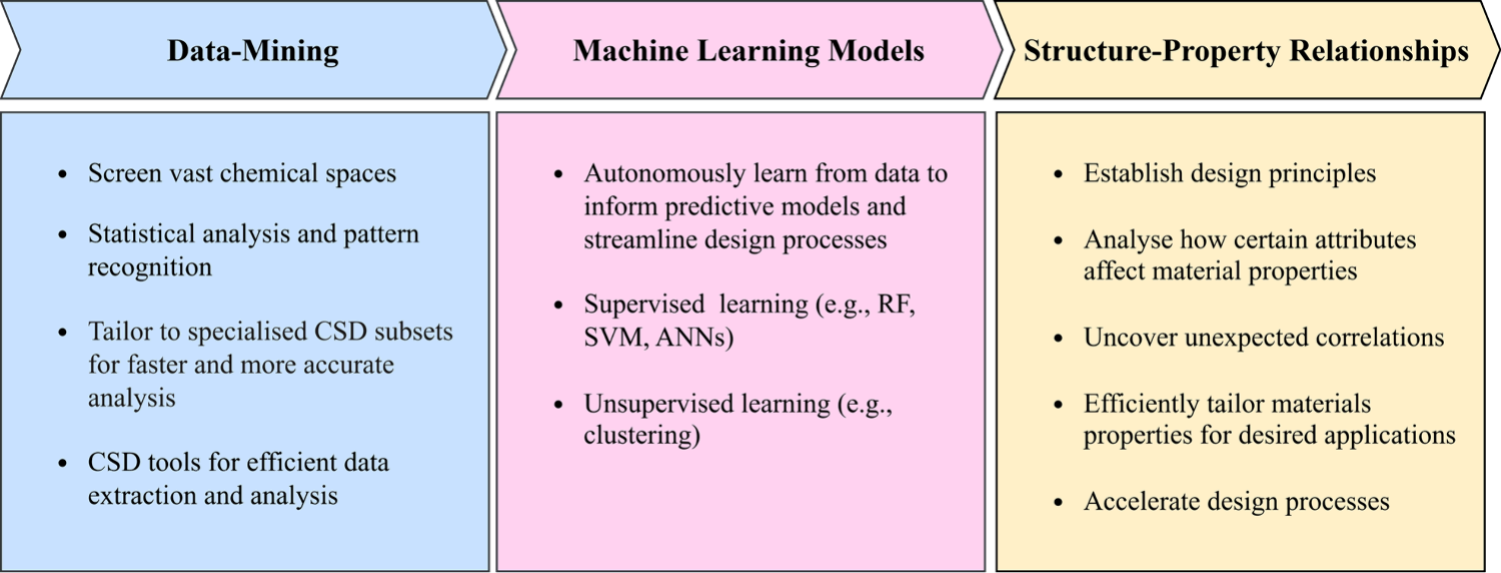
Summary of the use of the CSD and CCDC tools with data mining and machine learning (ML) techniques for identifying new structure–property relationships and enabling predictive materials design.

The CCDC software suite contains a collection of innovative tools that utilizes the data in the CSD to support these data-driven approaches. In addition to the ConQuest^2^ and Mercury^3^ software introduced above, the suite includes Mogul^4^ for accurately assessing molecular conformations, IsoStar^22^ for understanding crystal packing and intermolecular interactions, CSD-CrossMiner^5^ for pharmacophore-oriented queries of the CSD, and GOLD^6^ for predicting the binding of small molecules to targets from the Protein Data Bank (PDB). Finally, and again as introduced above, the CSD-Python API allows programmatic access to the CSD and many of these functions, enabling data searching, retrieval and analysis to be scripted and interfaced to other Python libraries for e.g. ML. Together, these tools allow the collection of experimental data in the CSD to be combined with state-of-the-art data science techniques to enable new approaches to materials design (Figure 1).

### 2.1 Data Mining

The general aim of data mining is to use statistical analysis and pattern recognition, such as classification and clustering algorithms,^17^ to identify relationships between the properties of known materials and chemical or structural descriptors (e.g., functional groups and crystal packing), potentially uncovering hidden correlations and yielding novel insights that can be used to target properties of interest.^18^ The extensive set of high-quality data in the CSD, and the comprehensive capability of the CCDC software suite, provides an ideal platform for this type of study.^18,23,24^

To better facilitate data mining, the CSD also includes specialized subsets of materials.^25,26^ The CSD-MOF subset is a collection of all the published MOF structures,^25^ and, paired with methods such as bond-type or cluster-type searches, facilitates efficient analysis of a wide range of MOF structures. A notable study utilizing the CSD-MOF subset is the data-mining approach by Moghadam et al., which developed a classification system for MOFs based on structural features such as secondary building units, surface chemistry, chirality and geometrical properties.^20^ This classification algorithm enables the rapid identification of structural features necessary for applications such as gas capture and storage.

The CSD-Drug subset is a collection of structures of approved drug molecules, allowing for data-mining studies to target structure–property relationships for active pharmaceutical ingredient (API, not to be confused with “application programming interface”) design.^26^ For example, Ma et al. mined selected structures from the CCDC-Drug subset and analyzed their lattice energies and intermolecular interactions to obtain an insight into packing arrangements and stability. The analysis revealed that phenyl groups contribute significantly to enhancing the lattice stability, and hence that optimizing aromatic interactions is crucial to designing stable drug forms. It also showed that dispersive interactions account for about 85% of the lattice energy, suggesting that optimizing van der Waals forces could be a useful design criterion for enhancing drug efficacy and stability.^27^

### 2.2 Artificial Intelligence and Machine Learning

Artificial intelligence (AI) and ML techniques aim to autonomously learn from a set of training data to develop predictive models that map features in the input data onto target output properties.^17,28^ As with data mining, ML models can identify new structure–property relationships, particularly when used with “explainable” AI methods to interpret model predictions.^29,30^ Another common use of ML is to “learn” the relationship between atomic or molecular descriptors and a property of interest in order to bypass expensive computational calculations.

ML algorithms are typically classified as “supervised” or “unsupervised” depending on the required input data. Supervised learning uses “labelled” data for model training. These methods tend to be more accurate, but require the data to be labeled (e.g., labeling molecules as drugs). Examples of supervised learning techniques include random forests (RFs), support vector machines (SVMs) and artificial neural networks (ANNs). Unsupervised learning techniques work on unlabeled data sets and tend to be less accurate, but may require less effort to prepare input data. Unsupervised methods include clustering algorithms and dimensionality-reduction techniques such as principal-component analysis (PCA), and can be used to identify previously hidden patterns in data without human intervention.^17^

Numerous studies demonstrate the potential of data-driven methodologies using the CSD for materials design. For example, a recent study by Nguyen et al.^31^ used CSD data and ML techniques to predict crystalline density and identify structure–property relationships relevant to energetic materials (high explosives). The authors employed a variety of molecular representations and input features (so-called “feature engineering”) and evaluated multiple ML algorithms. Message-passing NNs (MPNNs) were found to perform best for generalizing to chemically diverse and previously unseen materials, whereas RF and partial least-squares regression (PLSR) algorithms provided better insight into the importance of molecular features and identified a strong relationship between electronic and topological descriptors and density.^31^

## 3 Molecular Design

By drawing on the information on molecular conformations and intermolecular interactions in known crystal structures, the CSD and CCDC software enable the study of individual molecules and the design of new materials. In this section, we highlight some examples of where molecular design has been facilitated using these tools.

### 3.1 Drug Design

The pharmaceutical industry continues to be one of the most important industry sectors, with a > £40bn turnover and £5bn research and development (R&D) investment in the UK alone. Methods to assist with the rational design of APIs and solid form engineering, at all stages of the pharmaceutical pipeline from the design of new APIs for specific druggable targets,^32,33^ to understanding the solid-state chemistry that determines the processing steps required to produce a final drug formulation, are thus hugely impactful.

The ability to predict how a drug molecule will interact with a target protein, and subsequently its biological function, lies at the heart of drug discovery and development. In this context, the relevant parts of the CCDC software suite, such as CSD-CrossMiner^5^ and Genetic Optimisation for Ligand Docking (GOLD),^6^ play a crucial role. Both software packages provide data-driven insight into the drug–protein interactions that underpin pharmacological activity. With CSD-CrossMiner,^5^ pharmacophore-based queries are defined based on an abstract “model molecule” with the steric and electronic features required for interaction with a protein binding site. These are then used to search the CSD for matching small-molecule structures. GOLD^6^ provides a complementary approach of employing a genetic algorithm to predict how a molecule will bind to the target, taking into account conformational flexibility and possibly also user-specified constraints such as ensuring specific donor or acceptor group interactions are satisfied. GOLD can also provide some understanding of the impact that structural water molecules may have on the ligand binding site and docking. A number of recent studies have made use of both of these tools and interested readers are directed to the comprehensive review in ref.^32^

When considering binding, it is important to account for the statistical relevance of interactions to determine the probability of a binding event. This can be done using tools that extract and identify intermolecular interactions between a molecule or moiety of interest and another molecule or moiety, and evaluating the occurrence of these interactions in CSD structures.

While tools like CSD-CrossMiner^5^ and GOLD^6^ focus on predicting binding affinity and optimizing drug–protein interactions, Full Interaction Maps (FIMs)^34^ and IsoStar^22^ delve deeper into the molecular-level interactions that dictate the overall stability and efficacy of drug formulations. We briefly introduced IsoStar^22^ above as a tool for understanding crystal packing and intermolecular interactions, and FIMs^34^ provide comprehensive visual representations of the intermolecular interactions within a crystal structure. Understanding these interactions is pivotal for designing molecules that both bind effectively to their targets and exhibit desirable pharmacological properties.

**Figure 2:**
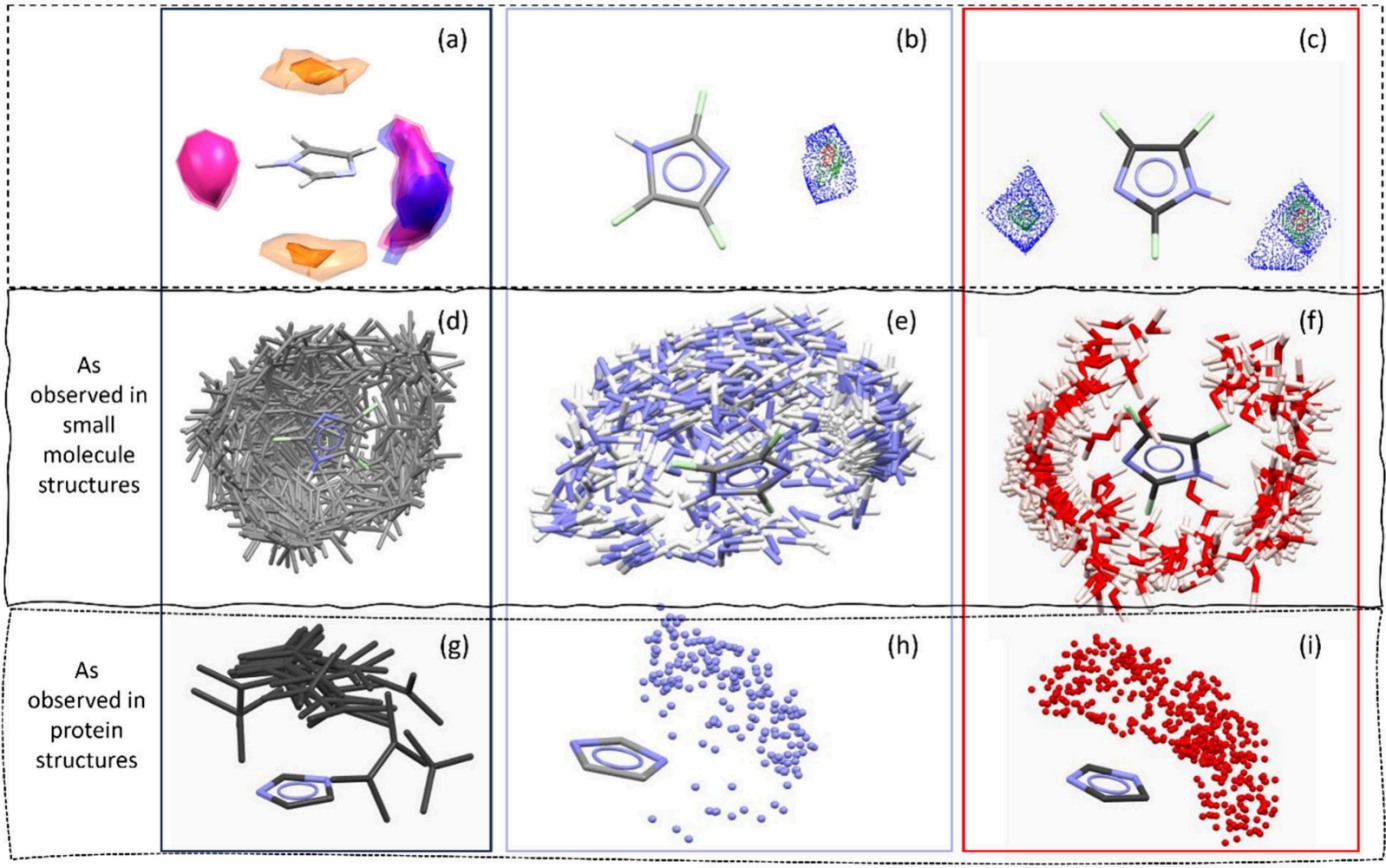
(a) Full interaction map^34^ of imidazole in small molecule structures (orange are interactions with aromatics, blue are interactions with N–H groups, and pink are interactions with O–H groups including in water and alcohols). (b)/(c) IsoStar^22^ analysis of the H-bonding of imidazole with (b) imidazole and (c) water. (d–i) Interactions observed in small molecules (CSD) (d–f) and in proteins (PDB) (g–i) with aromatics (d and g), N–H groups (e and h) and water molecules (f and i).

Figure 2 shows an example of this type of analysis for the small molecule imidazole. IsoStar^22^ analysis shows a stark difference between imidazole in an environment with other small molecules, and in a protein environment. The aromatic interactions in the small-molecule environment are truly random, with all possible orientations, whereas in the protein environment the π–π interaction mode dominates. The interaction with N–H groups is predominantly through H-bonding with the acceptor N, and again the positions of the N–H surrounding an imidazole are random in the small-molecule environment but very directional in the protein environment.

The interactions with water molecules are more interesting. In the small-molecule environment, water forms a rim around the plane of the molecule, whereas in the protein environment they are predominantly found above the plane of the aromatic ring. This type of insight into interactions in different environments is an important source of information for designing molecular structures that take the directionality of interactions into account, and may also help to understand solution and crystallization behavior. This analysis potentially also highlights the need for careful selection of data for data mining and ML studies to minimize the “background noise” from configurations that are not relevant to the environment being studied (c.f. Figure 2 (e) and (h)).

### 3.2 Catalysts

The CSD also serves as a foundational resource for AI-driven advances in catalyst development, mainly by providing comprehensive structural insight into both metal–ligand coordination and ligand geometries.^33,35−37^ This is exemplified by a recent investigation leveraging the CSD and associated tools for ligand and catalyst discovery.^38^ Initially, a high-throughput workflow and the CSD-CrossMiner^5^ tool were employed to mine the CSD and identify ∼32,000 potential ligands for the Cu(I)-catalyzed Ullmann–Goldberg reaction. ML models based on RF and SVM algorithms were then constructed to estimate the activation energy barriers for catalysts using these ligands, circumventing expensive computational modeling. These models were found to perform very well, with most of the predicted activation energies being within ±4 kcal mol^–1^, often taken as a threshold for “chemical accuracy”, of those obtained using “gold-standard” coupled-cluster methods. This study also uncovered important electronic ligand descriptors for catalyst design. Overall, this approach expedited screening and property prediction, with the promise of broad applicability across various chemical sectors including pharmaceutical process development. However, the reliance on semiautomated processes in this case highlights scope for further development toward full automation.^38^

It is also of note that CSD-CrossMiner^5^ has been extended to enable the study of other functional materials, including catalysts through the creation of “catalophore” queries analogous to the pharmacophore formalism,^38^ and host–guest chemistries by predicting the docking of guest molecules into metal–organic frameworks (MOFs).^39^ This showcases the flexibility and predictive power of these tools, but also the broad overlap between the materials design challenges in traditionally separate fields.

### 3.3 Perovskites

The structural information in the CSD has also been used to support the design of perovskites, a technologically important class of materials with potential uses as photovoltaics and solid-state lighting.^40^ For example, an ML study performed by Laref et al.^21^ sought to advance the design of the archetypal hybrid lead halide perovskites by elucidating the role of organic molecules in shaping the structure of the inorganic network. The authors used more than 600 structures from the CSD and >2,700 descriptors to develop an ML model capable of predicting whether a given organic amine would yield a perovskite-type structure with up to 88.65% accuracy. As part of this, they performed feature importance analysis to identify the 10 descriptors most relevant to hydrogen bonding, and they also established the number of ammonium cations as a critical criterion for determining whether a hybrid metal halide would adopt the target perovskite structure. This led to a design principle that the presence of a primary ammonium cation is crucial for synthesizing hybrid lead halide perovskites, irrespective of the dimensionality.^21^

### 3.4 Ferroelectrics

Another area where the CSD data and the CCDC suite of analytical tools have made significant contributions is in the design and discovery of molecular ferroelectrics. The quasi-spherical theory establishes that homochirality in molecular design can lead to molecules crystallizing in the five polar groups that enable ferroelectric properties.^41^ Attempts at using data-driven approaches to discover candidate ferroelectric materials have been somewhat limited by the scarcity and inconsistent quality of available data, as well as the difficulty in identifying appropriate descriptors. The comprehensive data available in the CSD, along with its suite of analytical tools, provides a means to address some of these issues.

An example that demonstrates this is the recent ML study by Ghosh et al.^42^ aiming to screen for potential ferroelectric materials using advanced ML techniques in conjunction with rigorously vetted data. Data on known molecular ferroelectrics was assembled and verified using the CSD. For small organic molecules, the selection process was further refined by excluding structures with an R-value value above 0.05 in order to ensure high data quality. Extensive feature engineering was performed, where molecular-level features were represented by 2D descriptors from the Molecular Operating Environment (MOE), while crystal-level features, such as atomic orbital energies, were implemented using the Matminer Python library.^43^ Several ML algorithms were assessed for their ability to accurately predict ferroelectric properties, in particular the magnitude of the spontaneous polarization. Among these, RF was selected for its performance with small data sets and its ability to effectively rank feature importance.^44,45^ Iterative refinement of both the data set and descriptors was performed to enhance the predictive accuracy, ultimately yielding a model based on ten critical descriptors and a revised data set that better balanced the representation of compounds with large polarization, achieving relatively accurate predictions with an RMSE of 1.84 μC cm^–2^. In addition to the high degree of predictive accuracy, this analysis also provided insight into the underlying structure–property relationships essential for the design of new ferroelectric materials.^42^

## 4 Solid-State Design

Crystal packing arrangements can change the physical properties in the solid state and have a large impact on processability, making strategies for solid form control extremely valuable. The pharmaceutical and agrochemical industries in particular, expend a great deal of effort and resources on solid form design and control. In this section, we explore the role of the CSD and CCDC tools in solid state design for three key classes of material, and highlight some challenges and opportunities for future development.

### 4.1 Polymorphism in Pharmaceuticals and Agrochemicals

Polymorphism is a hugely important consideration when developing a drug formulation, as evidenced by high-profile examples such as ritonavir (Norvir) and ranitidine hydrochloride (Zantac).^46^ Methods to investigate the likelihood of polymorphism for a new drug, as early as possible in the pharmaceutical pipeline, thus warrant significant R&D investment.

The CCDC Mercury software^3^ includes a number of tools that can be combined to provide a thorough assessment of the risk that an API may form other, previously undiscovered polymorphs. Hydrogen bond networks are often a key driver of polymorph stability. The Hydrogen Bond Propensity (HBP) tool^47^ aids in identifying and analyzing potential H-bond networks. The tool produces an H-bond chart showing the mean H-bond propensity against the mean H-bond coordination, providing a visual representation that effectively highlights structures with more probable hydrogen bonding networks. This tool also generates an H-bond propensity score table, which ranks networks based on their likelihood of occurring, with higher scores indicating greater probability. Finally, the HBP tool produces an H-bond coordination table that can provide further insight into the structural stability of different polymorphs by highlighting configurations where groups are optimally coordinated.

The Full Interaction Maps tool,^34^ introduced in Section 3.1 and Figure 2, extends the analysis beyond hydrogen bonds to encompass a wide range of possible intermolecular interactions that may influence the structures adopted by a target molecule and their relative stability. This tool creates a 3D visual representation of the probability of different types of intermolecular interactions using statistical data drawn from the extensive set of structures in the CSD, and can predict where functional groups from interacting molecules are most likely to be located relative to a target group. This information, when combined with 3D packing diagrams, allows the evaluation of whether a crystal structure satisfies the interactions expected for a particular molecule and/or conformation.

**Figure 3:**
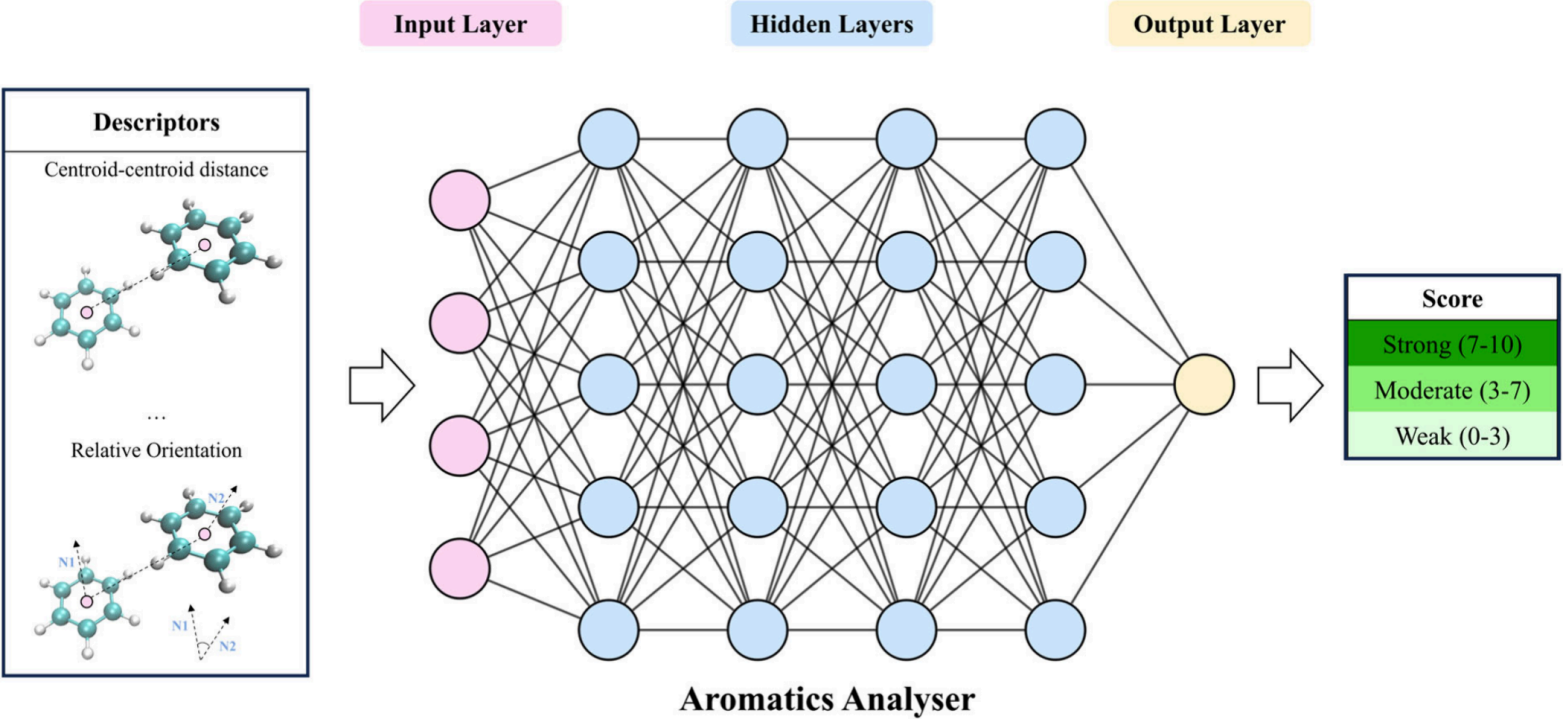
Schematic representation of the neural network-based Aromatics Analyzer tool in the CCDC Mercury software.^3^ Molecular descriptors representing the geometry of the aromatic interactions are fed into the input layer and are processed through hidden layers with rectified linear unit activation to yield an interaction strength score in the output layer, which then allows the interactions to be classified as strong, moderate, or weak.

Finally, the Aromatics Analyzer is the first example of a tool based on a trained NN, and provides a visual and quantitative assessment of the strength of aromatic ring interactions that may contribute to polymorph stability. This tool uses geometric descriptors such as atom–atom distances and plane–plane angles to represent the interactions between some types of aromatic ring pairs. It estimates the interaction energy through a network of hidden layers, which is then presented as a score from 0 to 10 allowing the interactions to be classified as “weak” (0–3), “moderate” (3–7), or “strong” (7–10) (Figure 3). The model is trained on data derived from density-functional theory (DFT) calculations and achieves an accuracy of 97% against structures containing aromatic functional groups from the CSD.

To illustrate the practical application of these tools, we take the example of para-aminobenzoic acid (PABA), a model drug known to crystallize in four forms, *viz*. α-PABA (CSD refcode: **AMBNAC07**), β-PABA (**AMBNAC08**), γ-PABA (**AMBNAC09**) and δ-PABA (**AMBNAC14)**. The α and γ forms are structurally similar and both feature cyclic acid dimer motifs packed along the [101] or [001] directions, and with the α form being slightly more stable. The β polymorph is centric and is stable at low temperature, transitioning to the α form above 14 °C.^48^ The δ form is a high-pressure form and features a similar head-to-tail motif as the β polymorph but in a noncentric structure.^49,50^

**Figure 4:**
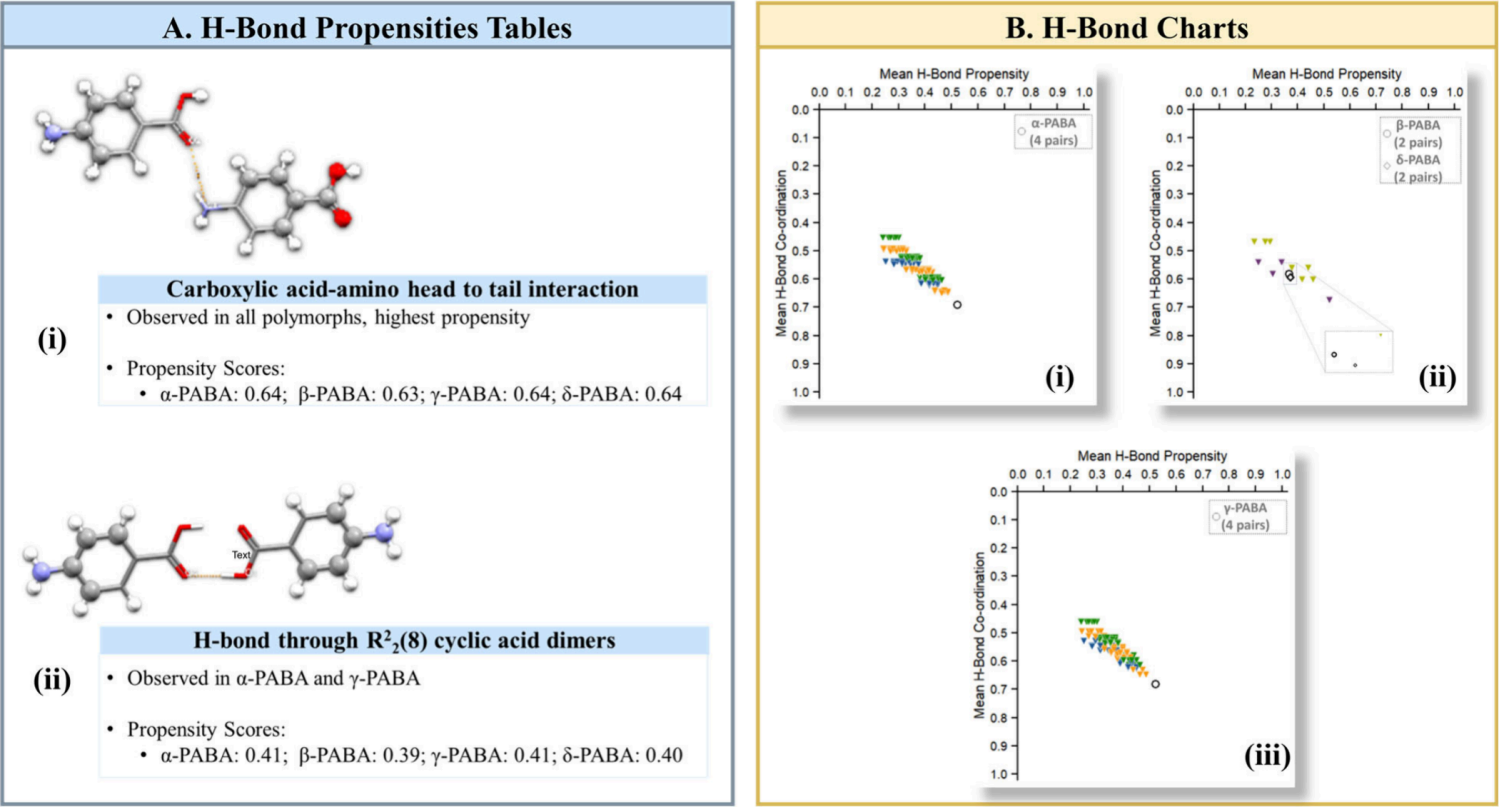
Hydrogen bond propensity (HBP)^47^ analysis of the four polymorphs of PABA: (a) Insights obtained from the H-bond propensity tables. (b) H-bond charts.

The HBP calculations in Figure 4 show that, while all four forms exhibit a strong propensity for carboxylic acid-amino group head-to-tail interactions (Figure 4 (a)(i)), there are notable variations across the four polymorphs that would influence their stability. The R_2_^2^(8) cyclic acid dimer interactions in the α and γ forms, albeit with lower propensity scores, indicate a secondary stabilizing mechanism that is absent in the β and δ forms (Figure 4 (a)(ii)). The position of the of the α and γ forms in the lower-right corner of the H-bond charts suggests they possess the most probable H-bonding network, whereas the location of the β and δ polymorphs near the middle of the charts points to less optimal hydrogen bonding (Figure 4 (b)).

**Figure 5:**
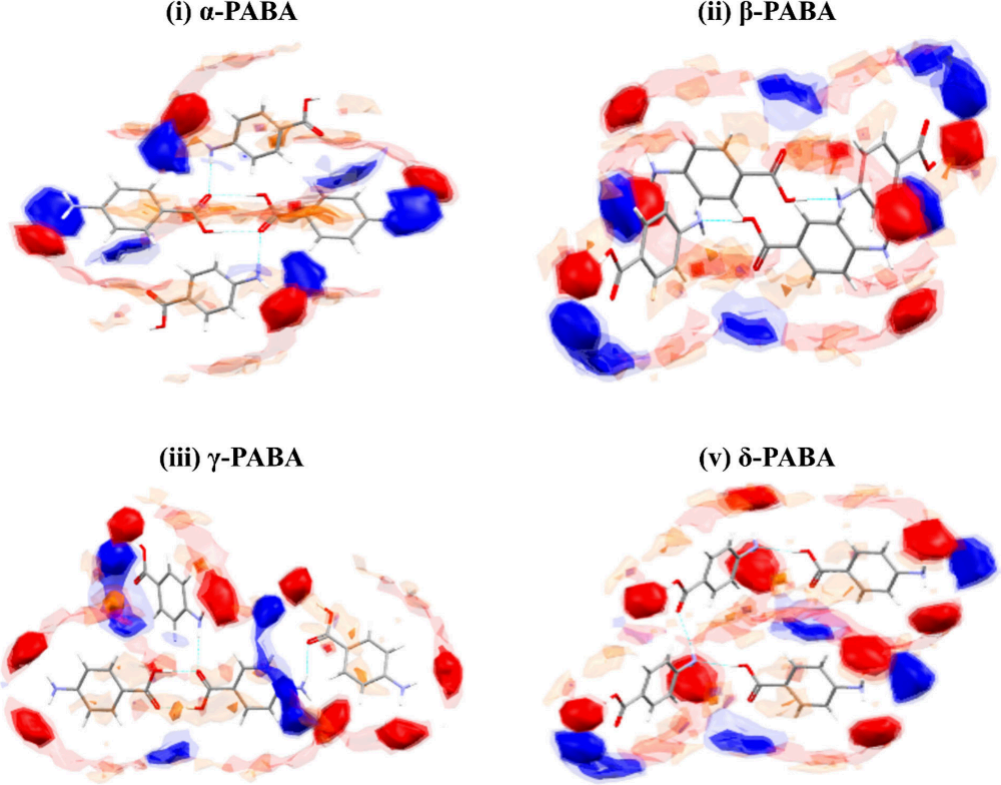
Full interaction maps^34^ for the four polymorphs of PABA. The map regions are colored based on the most probable interactions, *viz*. as a hydrogen bond acceptor (red) or donor (blue), or hydrophobic (brown). Dashed lines indicate H-bond contacts. The four polymorphs exhibit both different interactions and noticeably different interaction geometries.

The next step is to investigate the aromatic interactions in the polymorphs. Table 1 suggests mechanisms underlying the strongest interactions that are consistent with literature findings.^50^ For α-PABA, the stacking occurs through translation, resulting in two strong and three moderate interactions together with a large number of weaker interactions. These can be seen in the full interaction map (FIM) in Figure 5 (i), which shows hydrophobic regions (brown contours) over the carboxylic acid dimer. This distribution supports a robust network of aromatic interactions, likely contributing to the stability of the α form above the enantiotropic transition temperature of 14 °C.^48^ This is consistent with literature findings that α-PABA can be easily crystallized from various solvents above this transition point,^51,52^ suggesting a stable and strongly interacting molecular arrangement. The crystal structure of β-PABA is governed by the inversion symmetry in the stacking and displays a singular strong and several moderate aromatic interactions. The inversion symmetry may therefore lead to less effective packing, potentially explaining the documented difficulty of crystallizing β-PABA from nonaqueous solvents.^51,52^ Despite being the stable form below 14 °C, the aromatic stacking is not as favorable as in α-PABA, which may lead to a lower relative stability when not supported by specific solvent interactions. The FIM for β-PABA (Figure 5 (ii)) predicts a higher probability of aromatic interactions over the strong H-bonded acid-amine interaction than above the plane of the benzene ring. The γ-form also exhibits stacking through translation but, unlike α-PABA, presents an aromatic interaction profile with two strong, fewer moderate, and numerous weak interactions. This may indicate differences in how these interactions contribute to the relative stability. The literature suggests that the packing along the [001] direction in γ-PABA structure involves a unique arrangement of layers^50^ that may not optimize these aromatic interactions as effectively as in α-PABA, as predicted by the FIM in Figure 5 (iii).

**Table 1:**
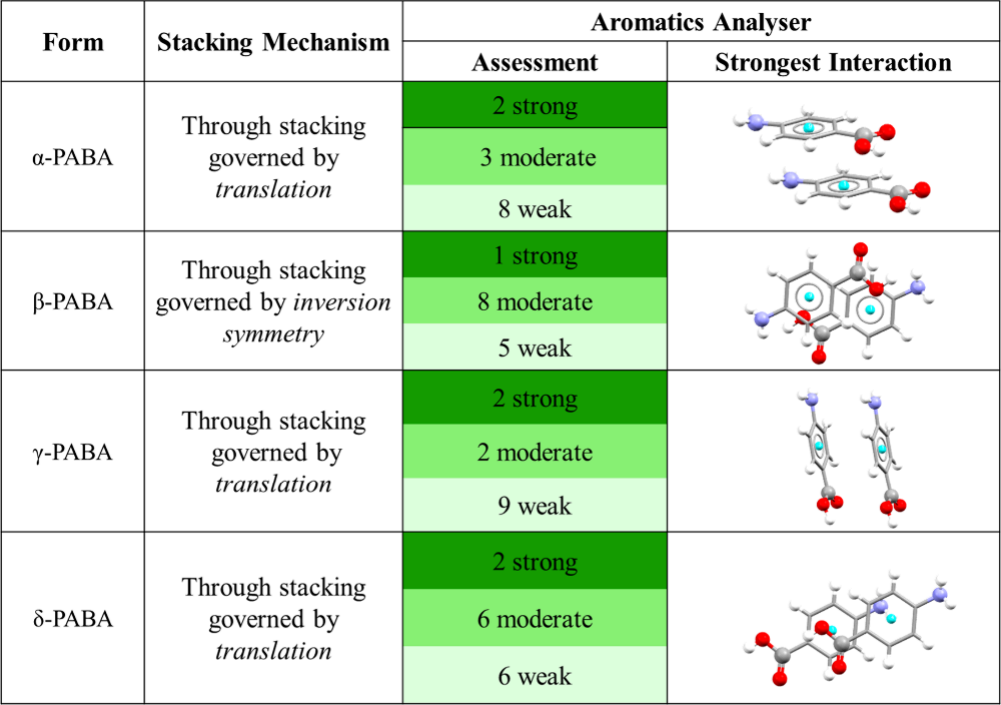
Comparative Analysis of the Aromatic Interactions in the Four Polymorphs of PABA Predicted Using the Aromatics Analyser Tool^a^

a The table categorizes interactions based on the mechanisms detailed in literature (translation and inversion symmetry),^50^ and lists their relative strengths (strong, moderate, weak) as determined by the tool. Visual representations of the strongest interactions in each form, again generated by the tool, are also shown.

The δ-form, which has similar stacking to γ-PABA, shows a balance skewed toward moderate interactions. This might be due to its noncentric crystal structure resulting in less robust aromatic interactions than in the centric forms, explaining its appearance under pressure rather than ambient conditions. The model the Aromatics Analyzer NN is trained on is based on gas-phase DFT calculations on benzene dimers, at centroid distances of 3.5–7 Å and a range of interaction angles between 0 and 90°, in a 15-molecule cluster.^19^ While the interactions in a high-pressure structure should fall within that remit, Wilson et al. identified that interaction energies increase with pressure to compensate for the loss of void space.^53^ This highlights a potential need to more carefully validate the NN model for high-pressure structures, as the training set used to construct the current model might not be representative of the interactions inthese structures.

The first application of combinatorial studies using the solid form informatics tools to assess the risk of polymorphism was reported in 2012, providing an initial exploration of this approach.^54^ A more comprehensive application to three example drug candiates was subsequently reported in 2015.^55^ The results of this study are highly significant as they show how statistical tools can be applied to harness the extensive molecular and structural information in the CSD to solve a fundamental, and potentially costly, challenge for the global pharmaceutical industry.

Expanding on these foundations, ML methodologies are emerging as powerful tools in the pharmaceutical sector, for initial screening processes to make an assessment of whether additional solid forms are likely to exist, perhaps revealing overlooked polymorphs, and to narrow down the chemical space to be investigated.^27,56^ One such example is the application of ML classification methods including RF and SVM by Hosni et al. to develop a “metaclassifier” approach to estimate the probability of polymorphism in organic molecules.^57^ Models trained using both the CSD^26^ and Drugbank^58^ data sets demonstrated impressive accuracy, particularly with a “prediction fusion” technique that achieved a remarkable accuracy of 91%. Moreover, validation of the model against 100 molecules excluded from the training data set revealed robust predictive capability.

### 4.2 Crystalline Porous Materials

Porous crystalline solids are a versatile class of materials for applications such as adsorption-based separations. The performance of such materials largely depends on their porosity, which makes the ability to predict and design solid forms with specific porosity highly desirable.^28^

Design of these materials has traditionally been approached through crystal structure prediction (CSP) followed by characterization of the porosity, which is computationally intensive and not well suited to high-throughput screening. As in earlier examples, ML techniques offer a route to overcoming this limitation by predicting key properties of interest without recourse to expensive modeling.^28^

García et al.^28^ used CSD data with ML techniques to establish correlations between the molecular structures of the building blocks of porous materials and their resulting porosity. Using RF ML models in conjunction with a comprehensive feature engineering process, including the development of porosity descriptors such as molecular pore exposure ratio (mPER) and molecular largest cavity diameter (mLCD), led to a novel approach to porous material design. The main finding was that porosity could be predicted to a significant degree of accuracy from the characteristics of the molecular building blocks, and this was confirmed quantitatively through a number of performance metrics. Important descriptors and structure–property relationships were also identified, such as a correlation between the mPER and material porosity descriptors such as gravimetric surface area, providing valuable general insights for the predictive design of porous materials and highlighting how understanding the intrinsic porosity of the molecular components can significantly accelerate the design and discovery of new porous materials.^28^

**Figure 6:**
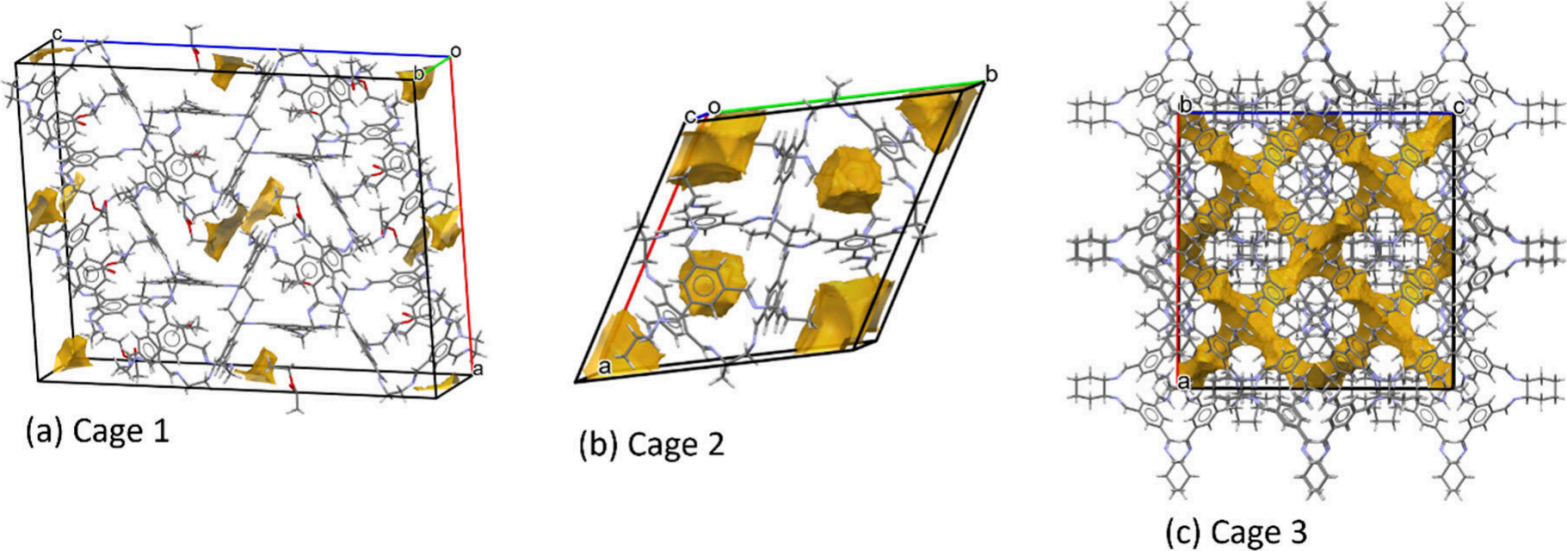
Analysis of three porous molecular organic structures using the void analyzer tool in the CCDC Mercury software.^3^ The analysis demonstrates that the pores in Cage 1 are not connected (a), whereas those in Cages 2 and 3 are (b)/(c).

The new porosity calculation tool in the Mercury software, an extension of the earlier void analysis tool, provides information about solvent accessible spaces and allows the use of helium and nitrogen probes to characterize pores identified using void analysis. This information can be used to compare a theoretical porosity value to trends in particle density measurements based on different methods and probe molecules. A nice illustration of this is the example porous organic cages designed by Tozawa et al.^59^ The three cages (Cage 1–3) have triangular pores by design, but the pore analyzer (Figure 6) predicts that Cage1 has no networked pores and hence would be unable to take up He, while Cages 2 and 3 do have networked pores. Cage 1 has a system volume of 2917 Å^3^, but only 3.65 Å^3^ is predicted to be accessible to a helium probe, whereas Cages 2 and 3 have system/accessible volumes of 1452/1271 Å^3^ and 6585/3414 Å^3^, respectively, explaining the finding from the gas adsorption studies by the authors that Cage 1 shows “porosity without pores”.

### 4.3 Metal–Organic Frameworks

Following on from porous crystalline materials, metal–organic frameworks (MOFs) are robust crystalline architectures that are almost infinitely tunable to produce different porosities and active sites for molecular adsorption, making them highly versatile candidates for applications from gas storage^60^ and separation^61^ to catalysis.^11^ The CSD hosts a large collection of MOF structures and provides a valuable data source for ML studies, in the context of both initial exploration and validation, facilitating the use of these techniques for the design and discovery of novel MOF materials.^10,16,18,20,36,62,63^ The notable work by Tang et al.^64^ focused on the rapidly screening MOFs for propane/propylene separation, which is a critical process in the petrochemical industry. This study used a combination of molecular simulations and RF ML algorithms trained using data from the “Computation-Ready, Experimental” (CoRE) MOF database.^65^ Through extensive feature engineering, a set of 254 descriptors capturing pore size, geometry and framework chemistry were extracted and used to train a model capable of accurately predicting adsorption capacities and selectivity for propane/propylene separation. To evaluate the transferability of the ML models, they were employed to screen MOFs from the CSD for C_3_H_8_/C_3_H_6_ separation. The predictions for the CSD MOFs showed good agreement with simulation results, suggesting that the ML models effectively transfer from the CoRE MOFs to CSD MOFs. Moreover, nine CSD MOFs were identified as having superior separation performance compared to the top-performing CoRE MOFs. This approach led to significant advances in the field of designing MOFs for gas separation by identifying key structural features such as pore size and geometry that are important for optimizing performance.^64^

## 5 Structure–Function Design

Building on the previous section, solid state design can be extended to create “functional” crystalline materials that, for example, respond to environmental stimuli with structural changes or changes in properties such as color. In this section, we discuss examples drawn from two families of materials, *viz*. multicomponent crystals and molecular switches, and highlight cases where the CSD and CCDC tools have been, or could be, used to provide insight into the underlying structure–function relationships.

### 5.1 Multicomponent Crystals

Multicomponent materials are crystalline compounds composed of two or more components in a specific stoichiometry, typically featuring directional interactions between molecules, and including salts and cocrystals.^66−68^ Multicomponent crystals provide a simple and often effective way to manipulate solid-state properties. They have applications to numerous fields including in the pharmaceutical industry, where they provide a means to optimize important physical properties such as dissolution rate and stability. This can have a range of benefits including increasing the solubility,^69,70^ and, by extension, bioavailability of drug molecules, improving chemical and physical stability,^71^ and optimizing bulk properties including crystal size and habit^72,73^ for processing steps such as particle filtering, flow, dispersion (for oral dosage forms) and compressibility (for tablet formation).^74,75^

The CCDC suite includes several tools dedicated to the rational design of multicomponent materials, which can be accessed through Mercury^3^ or the CSD-Python API. The Molecular Complementarity tool, developed with Fábián in 2009,^14^ provides a means to identify coformers likely to form a multicomponent crystal with a target molecule. The tool defines a selection of molecular descriptors that reflect the size, shape and polarity of the molecule, which are then used to assess the likelihood of complementary interactions with library of common coformers. Where complementarity is indicated, this suggests the potential to form a multicomponent material. As well as the coformer libraries available within Mercury, it is also possible for users to generate a bespoke library of coformer candidates for a more targeted study.^76^

There are numerous recent examples in the literature where the Molecular Complementarity tool has been used to guide experimental crystal engineering approaches to cocrystal formation,^76−79^ and the tool is frequently combined with other theoretical approaches. One recent study by Makadia et al. explored cocrystal design for the natural flavonoid apigenin and discovered six new cocrystal structures by combining the Molecular Complementarity tool with the H-Bond Propensity and H-bond energy analysis tools introduced in Section 4.1.^77^ In all cases, the new multicomponent solid forms showed enhanced dissolution compared to pure apigenin crystals, highlighting the utility of cocrystallization for tuning the physical properties of a target molecule. A validation test run by the CCDC highlights the need for further improvements to the tool, as it currently achieves an accuracy of up to 64%, but only when used for neutral molecules with molecular weights between 60–245 g/mol, and similar to which it was trained against. The accuracy further decreased with increasing drug molecular weights,^80^ highlighting an area for improvement in the future. A similar study using electrostatic potential surfaces as an alternative to the Molecular Complementarity tool for a large database of crystal coformers gave better results, and identified that phenolic groups generally act as better coformers than carboxylic acids, which tend to result in physical mixtures.^81^

Due to the complexity of the design space, ML techniques are increasingly being used to expedite and streamline the design and discovery of cocrystals.^23,82,83^ An innovative approach toward this goal is the one-class classification ML algorithm developed by Vriza et al.^84^ The ML model was trained on 1,722 molecular combinations extracted from the CSD, specifically focusing on cocrystals with π–π interactions. A key challenge was the inherent, and unavoidable, bias in the data set toward successful cocrystallizations, which was addressed through a comprehensive feature engineering process and carefully curated training data set. Dimensionality reduction was employed to streamline the data set, utilizing bidirectional concatenation to accurately represent molecular pairs. Molecular descriptors critical for understanding π–π interactions were identified and integrated into this process. The efficacy of the model was confirmed using 5-fold cross-validation, and demonstrated high accuracy in predicting potential π–π cocrystal formation. This study led to the discovery of two novel cocrystals, suggesting the approach holds promise for designing new multicomponent materials.

Expanding on this, more recent work constructed an attention-based NN screening tool, the Molecular Set Transformer, to prioritize molecular pairs that form stable cocrystals.^85^ Data was curated from all the available cocrystal data in the CSD and represented using fixed and learned representations. The issue of bias in the training set toward positive examples of cocrystal formation was addressed by employing an unsupervised, order-invariant approach to efficiently reconstruct input molecular pairs. A meticulously curated benchmarking data set from experimental reports was then used to evaluate the model, which outperformed or matched other ML and physical modeling methods. Overall, tools such as this further demonstrate the considerable potential of ML techniques to guide cocrystal design efforts, despite technical challenges such as the absence of negative data and the difficulty of constructing appropriate molecular representations.^85^

It is also important to recognize that materials sometimes crystallize as solvates, which are generally undesirable multicomponent systems. Several methodologies are being explored to overcome this challenge. For instance, Xin et al.^86^ employed RF and SVM ML algorithms trained on data extracted from the CSD to predict the solvate formation propensity of pharmaceutical molecules with a success rate of up to 86%. This type of predictive model is highly valuable for solid form optimization, as solvate formation can affect solubility, stability, and efficacy.

### 5.2 Molecular Switches

Switchable crystals, containing molecules that can be reversibly interconverted between two or more structurally distinct (meta)stable states on exposure to an external stimulus, require a carefully designed crystalline environment. For switching to proceed in a single-crystal-to-single-crystal manner, the host crystal matrix surrounding the responsive fragment must be able to accommodate an often significant amount of atomic or even molecular movement. For some processes, e.g. some photoswitching phenomena, each molecular rearrangement may proceed independently of other switching events, and in these cases the necessary atomic or molecular motions need only be accommodated on a local scale across the (usually very short) time scale of the photoexcitation process. In other cases, the switching may be cooperative and longer-range intermolecular interactions across larger regions of the structure must be considered. In all cases, the ability to make fast and visual comparisons between the starting (“ground state”) arrangement and the excited state structure can be highly informative, and considering that this is readily achieved using the tools in the CCDC software suite it is surprising that only a handful of studies have made full use of these tools to date.

For example, in pressure-responsive systems, where a structural change can be induced by the application of high (usually hydrostatic) pressure to the bulk crystal, volume minimization is the most important driving force for any pressure-induced transitions. As such, an understanding of the void space in the structure, and how this space evolves under applied pressure, can be hugely informative. As mentioned previously, Wilson et al. explored the importance of void space in pressure-responsive molecular crystals by analyzing a subset of 129 high-pressure structures extracted from the CSD.^87^ Using the CSD-Python API, the authors created a program to partition the volume changes under pressure into contributions from interstitial void space and changes to the bonding network. They then used this knowledge to understand which features are more easily compressible, allowing them to identify and explain the conditions under which a pressure-induced phase transition and/or crystal collapse would occur in a series of high-pressure studies.

For photoswitchable crystals, the “reaction cavity” concept, made popular by Ohashi et al.,^88,89^ can be thought of conceptually as the volume encapsulating the photoactive fragment in the crystal structure, and can be defined by determining the contact surface of the photoactive molecules (or atoms) within the cavity with the surrounding molecules in the crystal lattice. Using this definition, the reaction cavity can be visualized using the CCDC void space tools by simply deleting the molecules, or atoms, of the photoactive fragment (i.e., those that would be contained *within* the reaction cavity) then performing a void space calculation. This approach has been used in several studies of solid-state photoreactions, for example in linkage isomer crystals,^90−93^ to explain trends in photoreactivity including the maximum achievable excited state population fraction that can be accommodated in a particular structure.

The idea of removing key atoms or molecules of the active switching fragment and using void space analysis to obtain insight into material properties can be extended to the study of vapochromic switches. Work by Bryant et al. in 2017 explored the unusually fast vapochromic switching in a Pt(II)-pincer molecular crystal, which could be switched between red (water), yellow (dry) and blue (methanol) crystal forms on subsecond time scales.^94^ This fast switching behavior was explained using the void space tools in Mercury to visualize the space occupied by the small volatile organic compounds (VOCs) in the crystal, which clearly identified the pathways taken by the solvent molecules to enter or leave the structure.

Intermolecular interactions also play an important role in facilitating solid-state reactions. An easily understood example is the presence of hydrogen bonds to the switchable functional groups or fragments, as these relatively strong interactions must be broken, and often subsequently reformed, during conversion between the ground and excited state structures. Intermolecular interactions have been known to influence whether a photoreaction in a crystal can proceed to completeness, or even proceed at all, and the extent to which a given interaction can influence a reaction is often heavily dependent on the measurement temperature.^95,96^ CCDC tools such as full interaction maps, hydrogen bond statistics and hydrogen bond propensities all have the potential to be hugely informative in explaining how and why solid-state switching phenomena occur, and this is an area that we believe should receive more attention in future.

Finally, molecular materials are also being explored for neuromorphic computing applications to address some of the limitations of inorganic materials.^97^ Much like a biological neuron, molecular films made up of organometallic complexes can respond to different stimuli, through redox changes in the central metal ion, complex redox-induced electron transfer, isomerization and symmetry-breaking in the crystal packing. In light of the growing number of successful studies using the CSD and CCDC tools to identify key structure–property relationships, and to support the design of functional materials, we believe a similar approach could be used to accelerate the design of neuromorphic materials and/or to identify known complexes suitable for this application.

## 6 Surface, Interface and Particle Design

As established in the preceding sections, fundamental understanding of the structure–function relationships in crystalline molecular materials is key to successful translation from the lab scale to products. Among these, the surfaces and morphology of the particles formed during crystal growth, and the interfaces to other components in e.g. a formulation or device, are critical to process engineering and scale up but are often poorly understood.

The particle morphology is determined by the relative energies of the different crystal surfaces during crystal growth, and depends on the chemical functionality of the molecule, the crystal packing, and also potentially on environmental conditions such as the growth solvent in solution crystallizations. Once formed, the stability of a given particle shape depends on how the major surfaces, and the functionality exposed at these surfaces, interact with the environment under the storage conditions. The surface chemistry and particle morphology thus play a key role in determining a number of physical properties, including those that govern downstream processing during manufacturing.

Despite their importance, surfaces in general, and surfaces of crystalline organic materials in particular, are relatively poorly understood, and research in this direction can be regarded as a frontier in the materials design process. In this section we briefly describe the links between the bulk crystal structure, surface stability and particle morphology, and highlight some of the tools available in the CCDC software suite for studying surfaces and particle shapes.

### 6.1 Surface Energies and the Wulff Construction

The thermodynamically most stable particle shapes are those that predominantly expose surfaces with the lowest surface energy γ. Wulff stated, originally without proof, that the “height” of a surface extending outward from the center of a particle is proportional to the γ:
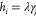
where the constant of proportionality λ depends on the number of molecules *n*. This so-called Gibbs-Wulff theorem is the basis for the widely used Wulff construction,^98^ which allows the equilibrium particle shape to be predicted from a knowledge of the surface energies. A Wulff construction requires a metric for the “morphological importance” (MI) of the crystal faces with Miller indices (*hkl*), which may be the surface energies *γ*_*hkl*_, or, more typically, the growth velocities *v*_*hkl*_, which in principle also account for the kinetics during the crystal growth.

Experimentally, the exposed surfaces of a crystal can sometimes be determined from X-ray crystallography, for example by face-indexing a single crystal on the goniometer or from the preferred orientation in a “powder” measurement collected without grinding.^99^ Surface energies can be measured using a variety of techniques,^100^ with more common ones being contact-angle^101^ and inverse gas chromatography (IGC) experiments.^102^ However, measurements can be challenging and may depend on the accurate determination of multiple parameters and the choice of model for interpreting the data.^103^ Growth velocities can be measured using optical techniques, such as observing crystal growth with purpose-built microscope setups or interferometry.^104,105^

### 6.2 The Bravais–Friedel–Donnay–Harker Model

Early work by Bravais attempted to relate the MI of different surfaces to the bulk crystal structure^106^ under the assumption that a higher density of material in a crystallographic lattice plane i indicative of stronger interatomic/intermolecular forces. This provided the basis for Bravais–Friedel–Donnay–Harker (BDFH) model for crystal morphology,^106^ where the MI of a surface is given by
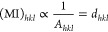
where *A*_*hkl*_ is the “reticular area”—the area of a crystallographic plane per node it intersects—and *d*_*hkl*_ is the spacing between lattice planes. A key feature of the BDFH model is that, since small {*hkl*} indicate large *d*_*hkl*_, low-index surfaces are more prominent. The BDFH model has the advantage of simplicity and of requiring only the crystal lattice parameters to predict particle morphology, but does not generally provide adequate predictions.

### 6.3 Surface Energies from Slab Models

In view of the complexities inherent to measuring surface energies, an alternative and widely adopted approach is to calculate the *γ*_*hkl*_ using atomistic modeling. The standard approach to calculate the *γ*_*hkl*_ for a surface with a given {*hkl*} is as follows. First, the bulk crystal is reoriented so the surface normal *n̂* lies along one of the Cartesian directions. Next, a vacuum gap is inserted along this direction to produce a 2D “slab” with two (typically identical) surfaces. Finally, the atomic positions are then relaxed, and the surface energy is calculated from
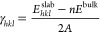
where *E*_*hkl*_^slab^ is the total energy of the optimized slab model with *n* molecules, *E*^bulk^ is energy per molecule of the bulk crystal and *A* is the area of each of the two surfaces.

This approach is most commonly used with quantum-chemical modeling techniques such as density-functional theory (DFT), although this need not be the case. The methodology is well developed, to the point where high-throughput approaches to determining *γ*_*hkl*_ have been developed and applied to elemental solids.^107^ However, a realistic slab model should generally be of sufficient thickness that the interior molecules are in a bulk-like environment, and the inherently larger unit cells of molecular crystals often require large models to achieve this, which can be problematic for DFT calculations.

### 6.4 Attachment Energies

A widely used alternative to the *γ*_*hkl*_ from slab models is to compute attachment energies *E*_*hkl*_^att^ (AEs) for adding a complete layer of molecules to a surface. The *E*_*hkl*_^att^ are defined as
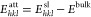
where *E*_*hkl*_^sl^ is the “slice energy” of a complete layer of molecules at the surface. The *E*_*hkl*_^sl^ can be obtained from energy calculations on slab models similar to those used to calculate the *γ*_*hkl*_, but with single layers of molecules and without relaxation. These two simplifications make computing the *E*_*hkl*_^att^ somewhat cheaper than the *γ*_*hkl*_.

The *E*_*hkl*_^att^ are a good approximation to the *v*_*hkl*_ and are inversely proportional to the MI.^108^ In contrast to the slab approach, attachment energy calculations are often performed using force fields, rather than DFT, with parametrized equations used to approximate the chemical interactions, although again this need not be the case.^109^ The attachment-energy approach with force fields is implemented in the popular HABIT codes^108^ and is widely used for predicting crystal morphologies.

### 6.5 Morphology Prediction Using CCDC Software

**Figure 7:**
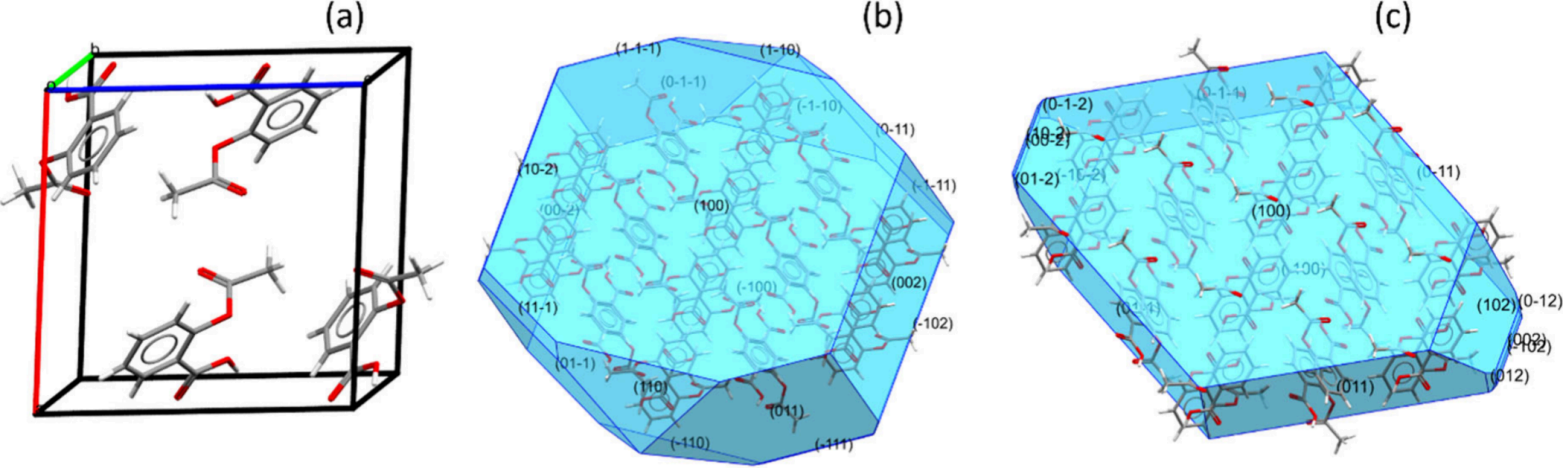
Crystal morphology prediction using the CCDC Mercury software.^3^ (a) Unit cell of aspirin Form I (CSD refcode: **ACSALA01**). (b) Morphology predicted using the BFDH method. (c) Morphology predicted using the Visual Habit software and attachment energy calculations with the Drieding force field.^110^

The CCDC Mercury software^3^ includes tools for predicting crystal morphology using both the BDFH and AE approaches. The AE tool currently uses one of three force fields, *viz*. the Dreiding II, Momany and Gavazotti models.^110−112^Figure 7 illustrates an example prediction of the morphology of aspirin Form I (CSD refcode: **ACSALA01**), and of the difference in morphology predicted by the BFDH and AE methods, which themselves differ significantly from the morphologies we observed in previous experiments.^113^

A new protocol by Spakman et al.,^114^ packaged into the CrystalGrower software,^115^ uses individual molecules as growth units and partitions the free energies of crystal growth, evaluated in a continuum solvation model. These energies are then coupled with Monte Carlo simulations to predict the growth of facets. This method allows the users to apply a bias to weight certain interactions more than others in order to replicate the experimental morphology. The existing morphology predictions available in Mercury could perhaps be improved with a similar approach, by enabling users to provide a bias to replicate a target morphology, which could then be interpreted using, for example, some of the tools for probing intermolecular interactions.

### 6.6 Challenges and Opportunities for the CSD and CCDC Software

Given the importance of improving our understanding of particle morphology, it is useful to consider how the CSD and CCDC software could contribute to and expedite current efforts. A study by Wilkinson et al.^116^ combined data from the CSD and in-house experimental data to construct ML models to predict the morphology of pharmaceutical crystals, which is crucial to solid form design, manufacturing, and to the pharmacological efficacy. The models were based on RF and NN algorithms and utilized a comprehensive molecular feature representation, encompassing both chemical descriptors and structural data, to correlate these features to particle morphology with a predictive accuracy of up to 87.9%. This study also identified some issues with the CSD data that are likely to pose challenges to this type of study, in particular limited access to the experimental details associated with CSD structures.^116^

In our view, the latter point highlights an important opportunity for the CCDC. Many of the crystal structures deposited with the CCDC include metadata on the sample morphology. By using natural-language processing to extract this information and compare it to morphology predictions using existing tools, it should be possible to better assess the accuracy of these techniques, and to highlight failures where further investigation could reveal new insight into the underlying mechanisms that determine the crystal morphology and/or identify improvements to the theoretical models. Face-indexing single crystals during data collection/solution is routine for accurately modeling absorption corrections, and this data could fairly easily be included in the CIF file (e.g., encoded as a set of relative surface areas to serve as MI descriptors for a Wulff construction). Doing so would, over time, provide a rich data set for studying particle morphology. However, the distinction between the mathematically predicted morphology and the crystal habit adopted experimentally in a given chemical environment should be considered to make this data set more applicable. Another possibility would be to also store low-resolution images of the crystals on the diffractometer, which could be used as input to ML models.

There are also challenges related to surface modeling and calculating the *γ*_*hkl*_ and *E*_*hkl*_^att^. For both types of calculation the accuracy with which different techniques can capture the inter- and intramolecular interaction energies is crucial. While a number of well-tested force fields are available for molecular solids (e.g., Drieding^110^), the simplified form of the potential-energy functions invariably means there will be some systems for which the interactions are not well described. On the other hand, the generalized-gradient approximation (GGA) functionals suitable for routine DFT calculations on molecular solids, and even more expensive hybrid functionals, tend to poorly capture intramolecular dispersion forces, leading to unrealistic lattice parameters,^117^ and it is easy to see how this might lead to errors in calculated *γ*_*hkl*_/*E*_*hkl*_^att^. The typical solution to this is to apply an additive dispersion correction, and these are a highly active development area.^118,119^ Low-level DFT functionals can also produce other issues, such as the “over-delocalization” of electron density leading to erroneous predictions of the relative energies of different conformations of the ROY molecule.^120^ Drawing a parallel with the many successful studies using ML for predictive design, an exciting recent development in this area has been exploiting ML to generate force fields from quantum-mechanical calculations,^121,122^ and such machine-learned force fields have the potential to strike the required balance between cost and accuracy for more ambitious modeling studies on molecular solids.^122^ For many of these techniques, an accurate sampling of the molecular conformational space is essential. This is typically generated through molecular dynamics or metadynamics simulations, but one has to be careful that the simulations adequately cover the full conformational space. The CSD could serve as a high-quality reference for this, either by identifying known conformations or for validating the “coverage” of a sampling process.

**Figure 8:**
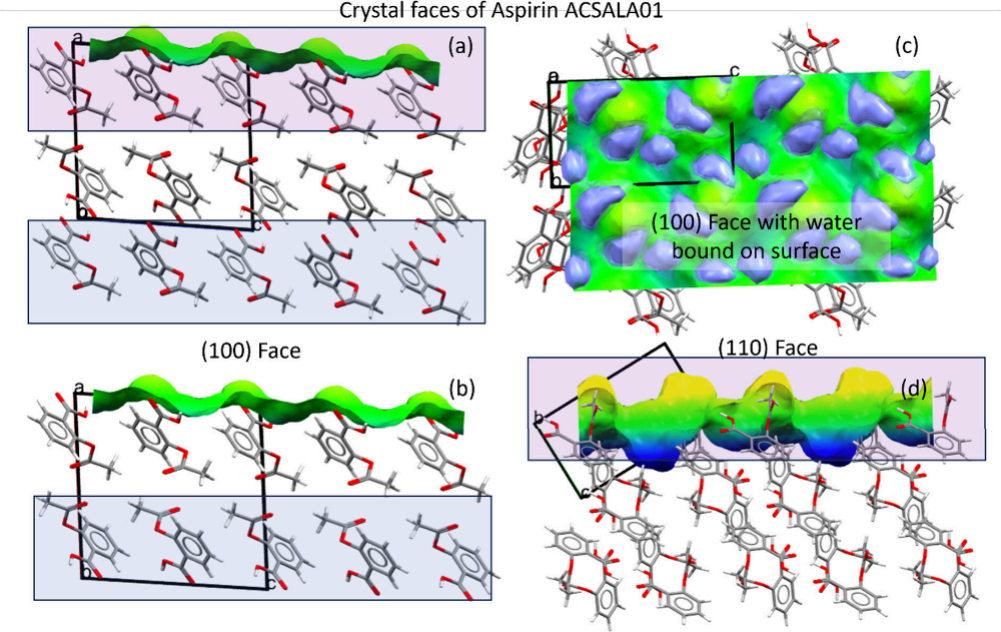
Modeling of the (100) surface of aspirin Form I (CSD refccode: **ACSALA01**). (a)/(b) Surface cuts of the (100) surface generated with the CCDC Mercury software^3^ showing two possible terminations exposing carboxylic acid (CO_2_H) and acetyl (Ac) functinality.^113^ (c) Full interaction maps generated for the (100) surface showing the likely interaction sites for water molecules. (d) Topography of the (110) surface.

A second challenge lies in the construction of surface models, in particular for determining *γ*_*hkl*_. Tools for preparing surface models that were designed around simple inorganic solids may not be programmed to ensure molecules remain intact when inserting the vacuum gap. Furthermore, for a given surface, multiple terminations, with different exposed functional groups may be possible, particularly for crystals of large, flexible molecules with multiple functional groups. From the perspective of particle properties, the latter is important, since the type of functional groups exposed at the surface (e.g., polar/nonpolar) will determine how the particles interact with their environment. We illustrate the second issue with the surface visualization tool in Mercury.^3^Figure 8 (a) and (b) show two cuts of the (100) surface of aspirin Form I.^113^ The carboxylic acid termination is calculated to have the lowest attachment energy among the various surface possibilities and is therefore always selected for the top surface.

The bottom surface of the slabs however can have both carboxylic acid (CO_2_H) and acetyl (Ac) terminations by changing the thickness parameter in the tool. Polarized Raman spectroscopy measurements found that both terminations of aspirin (100) could be obtained depending on the method of crystallization, with polar and apolar solvents favoring the hydrophilic CO_2_H and hydrophobic Ac terminations, respectively.^113^

Visualization of the (110) face also illustrates the complex topographies that can arise from the requirement to keep molecules intact (Figure 8 (d)), which would strongly affect the rugosity of the surface slice.

A third challenge, related to both of the first two, is how best to account for the influence of solvent on the surface energies. Molecular crystals are rarely produced by sublimation in vacuum and are more likely to be crystallized from solution. It is well-known that the growth solvent can influence the particle morphology,^123−125^ indicating that the interactions in solution can significantly impact the surface energies.

Methods for taking into account solvent interactions can broadly be divided into “implicit” and “explicit”. Implicit methods mimic the dielectric environment of the solvent. One example of this is the COSMO-RS approach^13^ for calculations on molecules, and a variation of this has been implemented for solids.^126^ COSMO is parametrized by a dielectric constant which accounts for the polarity of the solvent. By substituting the vacuum region in a slab model with a dielectric environment, implicit models can capture some solvent effects, but lack explicit interactions such as H-bonding that could, for example, preferentially stabilize polar surfaces. Explicit solvent models, as the name suggests, include explicit solvent molecules that can interact with the surfaces, and are more accurate but more expensive, particularly with methods such as DFT. A typical “middle ground” between these extremes is to include a layer of explicit molecules on the surface and treat the remainder of the vacuum gap with an implicit solvent model. As noted above, CCDC tools such as the interaction maps could be used to identify potential interactions and place solvent molecules, similar to the grid-based search for explicit solvent interactions built into the HABIT 98 tool.^127^ We suggest that the existing CCDC tools could be adapted into a robust workflow for generating and modeling surfaces with relatively little development effort.

## 7 Future Opportunities

Following the discussion in the previous sections, we identify some additional areas where we believe the scope of the existing CCDC tools could be extended to other materials design applications.

### 7.1 Predicting Mechanical Properties

Another key aspect of solid form design is the mechanical properties, which determine both potential applications and are important processing parameters. In general, materials must have suitable mechanical properties to withstand preparation, storage and application conditions. Small-molecule organics have historically not been considered for some applications due to their presumed plasticity, softness, and brittleness, but despite this their mechanical properties have proven useful in applications ranging from artificial muscles to flexible electronics.^97^ The excellent recent review by Awad et al.^128^ showcases the plethora of research underway on the mechanical properties of molecular crystals.

In this section, we present a forward look at possibilities for utilizing the CSD and CCDC software to incorporate mechanical properties into materials design. We consider “mechanical properties” in the loosest sense, and discuss both bulk and surface-related properties. For pharmaceuticals and agrochemicals, for example, this would include stability during downstream processing, such as reduced attrition (particle breakage) or punch sticking (crystals sticking to processing machinery). Recent work by the CCDC team explored the use of a new surface analysis tool^129^ to investigate the punch sticking properties of ibuprofen grown from different solvents, and identified differences in the electrostatic potential of the {110} faces due to the variable number of carboxylic acid groups exposed at the surface as the reason for the different punch sticking properties of different crystal morphologies.

For switchable materials, the ability to switch over a large number of cycles without e.g. buildup of stress is important - for example, multicomponent crystals based on diarylethenes can undergo photomechanical switching over a 1,000 times without showing signs of degradation, which makes them good candidates for photoactuators.^130^ Mechanical properties such as the bulk, shear and Young’s moduli define relationships between changes in internal forces (stresses) and deformations applied to the material (strains) using Hooke’s law. Using Voigt notation, the Lagrangian strain **ϵ** and stress **σ** are related through the second-order elastic constant matrix ***C*** according to^131^
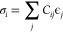
where in this notation the indices *i* and *j* each represent one of the pairs of Cartesian directions *xx*, *yy*, *zz*, *xy*, *xz* and *yz*. The number of independent *C*_*ij*_ depends on the crystal symmetry. For a stress-free crystal structure at equilibrium the elastic constants can be calculated from
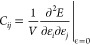
where *V* is the volume of the unit cell and *E* is the total energy. The *C*_*ij*_ can be calculated using finite differences, by applying small strains and calculating the changes in energy using force field models or quantum-mechanical methods such as density-functional theory.^131^ The CSD-Particle software suite included with Mercury^3^ already implements several force field models, which are used for the calculation of attachment energies as outlined in the previous section, which should make it relatively straightforward to calculate second-order elastic constants. However, as for surface energies these should be treated with caution until their accuracy has been carefully validated.

In most molecular materials, and in contrast to their inorganic counterparts, the elastic properties will be anisotropic due to low crystal symmetry and anisotropy in the intermolecular interactions. Lubomirsky et al. computed face-specific Young’s moduli for crystalline amino acids using dispersion-corrected DFT and obtained results in good agreement with experimental measurements.^132^ They were able to attribute the large moduli of some of these crystals to underlying charge-assisted intermolecular interactions, and, while many molecular materials would be mechanically “softer” than amino acids, this suggests designing molecules or multicomponent crystals that would show similar interactions could provide mechanical hardness and, for example, optimize resistance to cracking during property cycling. This example hints at how, as in previous sections, the CSD and CCDC software could be used to establish the structure–function relationships relevant to mechanical properties.

So-called “highly tough” materials with an extended plastic zone, where the structure is capable of absorbing large amounts of energy per unit volume and is thus resistant to cracking, are one potential target, addressing a common assumed problem with molecular solids. Tools to identify tough materials, and to identify new structure–function relationships to design new molecules and supramolecular architectures, in our view represents a logical first step. Existing CCDC tools, such as the tool in Mercury^3^ for identifying “slip planes” and the H-bond analysis tools, could be used to identify structural features that correlate to brittleness or plasticity.

While toughness is a bulk property, hardness is considered a surface property.^128^ For some applications, solid forms of a material must be interfaced to other materials, for example metals or semiconductors in optoelectronic devices, or organic or bioorganic materials in pharmaceutical formulations. As discussed in the previous section, establishing and controlling the nature of the surfaces and interfaces is an important challenge, and understanding their impact on mechanical properties is a key part of this.

Recently, defects induced by mechanical stress or the removal of water from dihydrates of the drug molecule carbamazepine, leading to stacking faults in one direction and twinned domains and grain boundaries in the others, have been identified using transmission electron microscopy and linked to strongly anisotropic mechanical properties.^133^

This behavior is also seen in ferroelastic materials, the mechanical equivalent of ferroelectricity and ferromagnetism whereby a material exhibits a spontaneous strain that can be switched between two or more stable orientations. Switching occurs through the formation of twin domains, but, uniquely, the associated grain boundaries can spontaneously heal following the switching rather than propagating through the material as cracks. Twinning is a complex phenomenon, and we leave a detailed description to other literature.^134^ However, structural features that indicate a propensity for twinning have been identified. First, it tends to be more prevalent in crystals in low-symmetry and/or polar spacegroups. The generally lower symmetry of molecular crystals compared to inorganic materials results in a generally higher tendency to twin and to exhibit more complex twinning behavior. Klassen-Neklyudova further establishes some “rules of thumb” for mechanical twinning,^135^ for example that an undeformed plane cannot be normal to a 4-fold axis in a mirror twin and cannot coincide with a 2-fold axis in an axial twin. This suggests it may be possible to identify features in bulk structures that predicate potential twinning mechanisms and that can be used to infer the associated transformations (twin laws). The structure–function relationships may be complex, and we therefore suggest this as a candidate for an ML study.

In addition to ferroelasticity, some crystals exhibit bending and twisting in response to environmental stimuli such as external stress, impurities, or to accommodate internal geometric frustrations during crystallization. Twisting occurs through loss of translational symmetry and is characterized by a pitch length *P*, associated with a 180° rotation of the crystal, given by
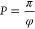
where φ is the twist per unit length. In a similar manner to the slip plane tool, which considers only translational symmetry, a “twisting propensity” tool that assesses H-bonding and aromaticity around a screw axis could provide a means to identify spiral growth and potential mechanical twisting. The ability to grow slab/slice models including these features would also enable the impact of e.g. surface reconstructions in nanosized particles on this behavior to be assessed, and for the impact of the behavior on particle properties to be analyzed.

### 7.2 Exploiting Advances in Crystallographic Data Collection for Future Predictive Capability

In the previous section, we touched upon the possibility of improvements to crystallographic data collection to support future predictive capability. To keep Olga Kennard’s pioneering vision alive, data collection and curation must keep up with technological advances in the field. These include, but are by no means limited to, improvements in spatial and temporal resolution, and in the resolution of the momentum transfer of diffracted photons.

More widespread access to high-resolution data collection means that charge density analysis for molecular materials^136,137^ might be considered more routinely. Achieving good data statistics to 0.8 Å *d*-spacing is “good” when using the independent atom model (spherical atomic form factors), while for nonspherical atom refinements a *d*-spacing of 0.8–0.7 Å should be a bare minimum to resolve features such as lone-pairs around the atoms. Experimental charge density analysis, to model the electron density as multipoles in the form of s, p, d orbital constructs, requires good data statistics to a *d*-spacing of 0.4 Å - 0.5 Å as a minimum.

Among other things, charge density analysis could allow for interesting comparison with DFT and other quantum-mechanical calculations, from which electron densities are readily available, and, when paired with topological analysis methods^138−141^ could provide a rich data set for characterizing interatomic and intermolecular interactions. This type of insight has, for example, been used in conjunction with ML to develop highly accurate and transferrable force fields including geometry-responsive multipolar electrostatics,^142^ which can be applied to calculations on molecular solids.^143^

The combination of high-flux X-ray sources and highly sensitive photon-counting detectors has enabled time-resolved diffraction studies, where structural changes in response to environmental stimuli can be examined with atomic resolution on subsecond time scales.^144,145^ Using existing or new tools to analyze these time-resolved data sets could, for example, reveal new structure–property relationships to guide the design of responsive crystalline solids such as those discussed in Section 5. The latest X-ray detectors can output the time and location of individual photon measurements as a continuous data stream, as opposed to the time-binned images from traditional detectors,^144,146^ which will make this type of study far easier by allowing time resolution and data quality to be balanced after collection.

Finally, exploiting improvements in momentum resolution to routinely collect total scattering information alongside the Bragg scattering could provide a means to study disorder and to explore phenomena such as defects. Diffuse scattering methodology for studying inorganic materials is fairly well established, but developing the technique for molecular materials brings a number of challenges and is another frontier topic in structural science. For interested readers, a recent review^147^ provides a good introduction to this topic.

We note that in all three cases careful consideration should be given to ensuring complete metadata is deposited alongside structures (e.g., the conditions under which measurements were made in a series of time-resolved structures). Indeed, as discussed in the previous sections, improving metadata collection for routine crystal structure determinations, e.g. by capturing information about the sample preparation and morphology, would make the CSD data more useful for a number of current and future applications. Another point for consideration is whether it is useful, or indeed feasible, to capture raw data such as diffraction images alongside processed data and solved structures. This is increasingly seen as good research practice, and would, for example, allow for reanalysis in the future if and when improved data treatments become available, but for some studies will inevitably require considerably more storage than the processed data.

## 8 Conclusions

This perspective has demonstrated how the Cambridge Structural Database, which began as a pioneering means to collect and store crystallographic data in a standard format, has evolved into an indispensable resource for predictive design of a wide range of molecular materials. The CSD is now firmly established as the repository of choice for small-molecule crystal structures, and, with 1.25 million structures and counting, ranks among the most comprehensive data sets in the materials sciences. The comprehensive and actively developed CCDC software stack, which provides search, visualization, advanced analysis and automation capability to exploit data in the CSD, makes it possible for academia and industry to address challenging questions at the forefront of research across a wide range of fields.

Molecular materials design is a complex multiscale problem, spanning the design and synthesis of molecules, the design of solid forms and exploitation of solid-state structure–function relationships, the control of particle properties and interfaces, and optimization for downstream processing and scale-up. The synthetic diversity of molecules and the scope for solid form and particle engineering together produce an almost unlimited design space. Molecular materials are thus an inherently challenging area, but, as the many applications highlighted in the case studies in this perspective demonstrate, one with limitless opportunities.

Many of these challenges are well-known to the pharmaceutical and agrochemical industries, and the role of these industries in shaping the CCDC software, including through the Crystal Form Consortium, is clearly evident in capabilities such as CSD-CrossMiner^5^ and GOLD.^6^ The opportunities presented by the strong interest in metal–organic frameworks, and the clear route to controlling function through the structure of the metal clusters and ligands and the 3D connectivity in the solid state, highlights another textbook example of how the data in the CSD can, with the right tools, be a powerful resource for materials design. Building on this, the addition of “catalophore” queries to extend the capabilities of CSD-CrossMiner^5^ to catalyst design shows how innovations in one field can also benefit others. In this vein, we have identified several topical research areas we believe the CSD and CCDC software are well positioned to address, including functional materials, surfaces and interfaces, and mechanical properties. Of these, some will require straightforward repurposing of existing tools, some will require implementing new functionality, and others may require more foundational changes to the way structural data is collected, processed and archived in the CSD.

In summary, the central role of the CSD and CCDC software in contemporary molecular materials science demonstrates that Olga Kennard’s original vision has very much been realized, and we are confident that her legacy will continue to enable new science well into the future as the CCDC continues to refine its data collection and curation strategy and leverage the CSD to develop new tools for its diverse user community.

## Glossary

Term: Definition
Data-Driven: Relying on the collection or analysis of data to guide decisions and strategies.
Pharmacophore: The essential set of structural features in a molecule that determines its biological activity and ability to interact with a specific target.
Data Mining: The process of identifying meaningful correlations, patterns and trends by analyzing large amounts of data.
Artificial Intelligence (AI): The simulation of human intelligence in machines that are programmed to perform tasks such as learning and reasoning.
Machine Learning (ML): A subset of AI where algorithms are used to enable computers to learn from and make predictions or decisions based on data, without being explicitly programmed for specific tasks.
Classification Algorithm: An algorithm used to assign labels or categories to input data based on its features. The algorithm learns from labeled training data and applies this knowledge to classify new, unseen data.
Clustering Algorithm: An algorithm used to group similar data points into clusters based on their features. Unlike classification algorithms, clustering algorithms do not require labeled data.
Features/Descriptors: Quantitative or qualitative characteristics/representations of a molecule or compound used to describe its properties and behavior.
Feature Engineering: The process of selecting, modifying, and transforming raw data into features that can be effectively used by ML models. This involves crafting features that enhance the ability of the model to learn and make accurate predictions by providing meaningful and relevant input from the original data.
Structure–Property Relationships: The links between the molecular structure and the physical or chemical properties of a material.
Supervised Learning: ML techniques using labeled data for training, including algorithms like random forests, support vector machines, and artificial neural networks
Random Forest (RF): A ML method that uses a large number of small decision trees, called estimators, that each produce their own predictions. RFs can be used for a variety of tasks including regression and classification.
Support vector machine (SVM): A supervised ML algorithm that can classify data by finding the hyperplane that best separates different classes.
Artificial Neural Network (ANN): A computational model inspired by the structure and function of the human brain. ANNs are composed of layers of interconnected nodes, or “neurons,″ where each node processes and transmits input data to subsequent layers. The network learns to recognize patterns, make predictions, and improve its performance by adjusting the connections (weights) to minimise prediction errors.
Unsupervised Learning: ML technique that works on unlabeled data sets, includes clustering algorithms and dimensionality-reduction techniques like principal component analysis
Message-passing NN (MPNN): A subclass of graph neural networks (GNNs) that integrate multiple GNN types into a unified framework. MPNNs model complex interactions between nodes and edges in a graph through iterative message-passing, where each node exchanges information with its neighbors. This process aggregates local details and computes global representations to capture the structure and relationships within the graph.
Partial least-squares regression (PLSR): A statistical method used to model the relationship between a set of predictor variables and a set of response variables by projecting them into a lower-dimensional space. PSLR is used to identify important molecular features for describing material properties.
Principal Component Analysis (PCA): A statistical method that transforms high-dimensional data into a lower-dimensional form by identifying the directions along which the variance of the data is maximized.
Dimensionality Reduction: A technique used to reduce the number of variables under consideration while retaining as much relevant information as possible. One such method is PCA.
Genetic Algorithm: A computational method inspired by natural selection, used to solve optimization problems by evolving solutions with biomimetic processes over optimisation iterations.
High-Throughput Workflow: An automated process that allows for the rapid testing and analysis of a large number of data points.
Coupled-cluster method: A computational technique used to calculate molecular electronic structure by introducing interactions among electrons within a cluster (e.g. electron pair interactions) and allowing the wave function to include all possible couplings among these clusters.
Catalophore: The set of structural features in a molecule that are crucial for its catalytic activity, facilitating the interaction between the catalyst and its substrate.
Quasi-Particle Theory: A framework that simplifies complex many-body systems by using effective particles, known as quasi-particles, to represent collective excitations and interactions within the system.
Molecular Operating Environment (MOE): A software suite for molecular modeling, computational chemistry, and drug design, offering tools for visualization, modeling, and data analysis.
Metaclassifier Approach: A machine learning technique that combines the predictions of multiple base classifiers to improve overall accuracy. Metaclassifiers function by training a classifier to make final predictions based on the outputs of several other models, leveraging their collective strengths and mitigating individual weaknesses.
Crystal Structure Prediction (CSP): The prediction of the crystal structures of solids using only knowledge of the constituent atoms or molecules.
Cross-Validation: A statistical technique used to evaluate the performance and generalizability of a predictive model. Cross-validation partitions a data set into multiple subsets, training the model on some while testing it on the others, and repeating this process multiple times to assess the performance of the model across different subsets of the data.
Density-functional theory (DFT): A theoretical framework that provides a quantum mechanical description og s molecule or solid using the electron density instead of the many-body electronic wavefunction.
